# Manipulation of the Unfolded Protein Response by Intracellular Bacterial Pathogens: Mechanisms of ER Hijacking and Therapeutic Implications

**DOI:** 10.1096/fj.202503547R

**Published:** 2026-01-17

**Authors:** Enhui Dai, Dongjie Sun, Yanxiao Zhao, Mengtao Zhang, Yifan Wu, Jiabo Ding

**Affiliations:** ^1^ Institute of Animal Science, Chinese Academy of Agricultural Sciences Beijing China; ^2^ College of Veterinary Medicine Shandong Agricultural University Taian China

**Keywords:** bacterial infection, effector proteins, endoplasmic reticulophagy, immune response, therapeutic strategy, unfolded protein response

## Abstract

The unfolded protein response (UPR) is a cellular stress response mechanism that maintains endoplasmic reticulum (ER) homeostasis through three signaling pathways mediated by IRE1α, PERK, and ATF6 sensors. While UPR's role in viral infections has been well documented, recent studies indicate that intracellular bacterial pathogens have evolved specific mechanisms to hijack UPR signaling for survival and replication. This review examines UPR manipulation strategies employed by major bacterial pathogens, including *Brucella*, *Mycobacterium tuberculosis* , *Legionella*, and *Salmonella*. These pathogens utilize effector proteins that target specific UPR components: *Brucella* effectors VceC, BspB, TcpB, and BspL interact with ER chaperones and ERAD machinery; *M. tuberculosis* proteins Rv0297, ESAT‐6, HBHA, and CdhM disrupt calcium homeostasis and alter ER morphology; *Legionella* Lpg0519 activates atypical ATF6 signaling; and bacterial toxins including cholera toxin bind IRE1α structural motifs for pathway activation. The molecular basis of UPR manipulation includes direct protein–protein interactions, calcium signaling interference, ER morphological disruption, and transcriptional program modulation. Bacterial hijacking of UPR pathways affects ER‐phagy processes and host immune responses, facilitating intracellular survival. UPR pathway components serve as potential targets for host‐directed therapy against persistent and drug‐resistant infections. Small molecule modulators targeting IRE1α kinase activity, PERK inhibitors, and ATF6 pathway regulators may complement conventional antimicrobial approaches. Characterization of these host‐pathogen interactions provides insights for developing therapeutic strategies that target bacterial dependencies on cellular stress responses.

## Graphical abstract

This review illustrates how intracellular bacterial pathogens—such as *Brucella*, *Mycobacterium tuberculosis* , and *Legionella*—secrete effector proteins that specifically target the IRE1α, PERK, and ATF6 branches of the unfolded protein response (UPR) to hijack ER stress signaling. By doing so, they remodel endoplasmic reticulum (ER) homeostasis, modulate ER‐phagy, and manipulate host immune responses to establish a replication‐permissive intracellular niche. The review also discusses host‐directed therapeutic strategies aimed at targeting the UPR to counteract bacterial exploitation.

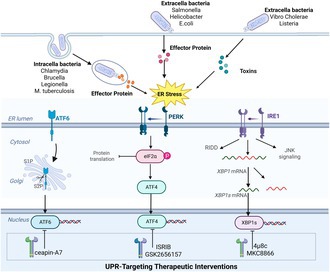

## 1 Introduction

The endoplasmic reticulum (ER) is a crucial cellular organelle responsible for the synthesis, folding, modification, and transport of proteins, as well as the primary base for the biosynthesis of membrane lipids and cholesterol [1, 2]. It is estimated that nearly one‐third of all eukaryotic proteins are synthesized in the ER and subsequently enter the secretory pathway [3]. The ER orchestrates the processing and proper folding of secretory and membrane proteins, guiding them into their correct three‐dimensional conformations with the assistance of a specialized oxidative environment and molecular chaperones, such as heavy‐chain binding protein (BiP) [4]. Once correctly folded, these proteins are transported from the ER in vesicles to the plasma membrane, various organelles, or secreted outside the cell, thereby maintaining cellular homeostasis. However, when the demand for protein folding exceeds the ER's capacity, unfolded or misfolded proteins accumulate, triggering a condition known as endoplasmic reticulum stress (ERS). ERS activates a series of adaptive responses, including the unfolded protein response (UPR), ER overload response (EOR), and the sterol regulatory element‐binding protein (SREBP) pathway [5].

While the UPR's role in viral infections is well‐documented, its exploitation by intracellular bacterial pathogens represents a rapidly evolving and critically important frontier in host‐pathogen interactions. Emerging evidence indicates that bacteria do not merely induce ER stress as a byproduct of infection; instead, they have evolved sophisticated arsenals of effector proteins to actively hijack UPR signaling pathways, transforming a host defense mechanism into a tool for their own survival and replication [6, 7, 8].

This review synthesizes the current understanding of how key bacterial pathogens, including *Brucella*, *Mycobacterium tuberculosis* , *Legionella*, and *Salmonella*, precisely manipulate the IRE1α, PERK, and ATF6 pathway of the UPR. We delve into the molecular tactics employed, their impact on ER‐phagy and host immunity, and crucially, explore the promising therapeutic landscape of targeting these hijacked pathways to combat persistent and drug‐resistant infections.

## 2 The Unfolded Protein Response Network

### 2.1 UPR Sensor Architecture and Function

**FIGURE 1:**
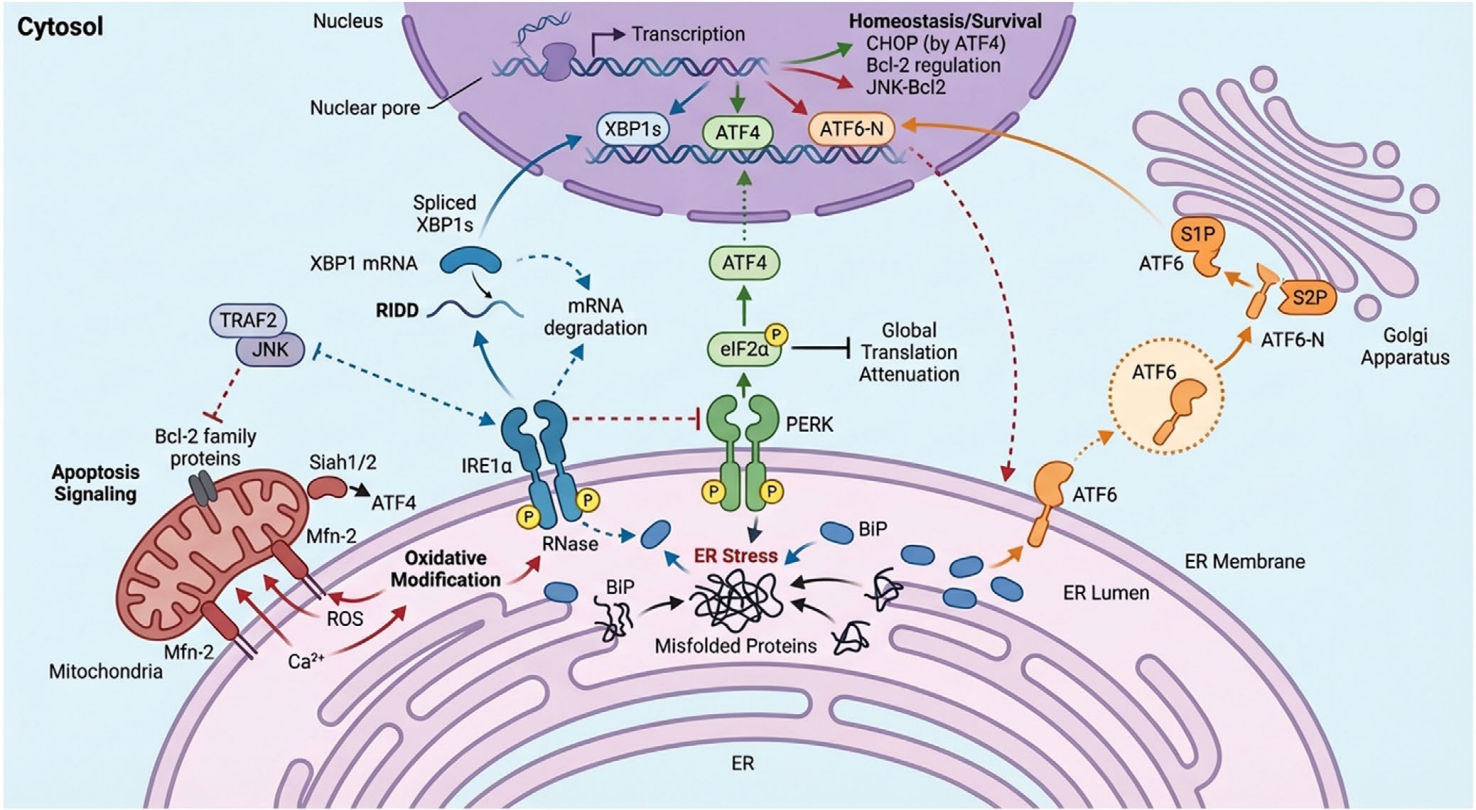
Schematic overview of the unfolded protein response (UPR) signaling network. Accumulation of misfolded proteins in the ER lumen leads to dissociation of the chaperone BiP from the three principal ER transmembrane sensors—IRE1α, PERK, and ATF6—thereby activating their downstream signaling pathways. Activated IRE1α undergoes autophosphorylation and induces XBP1 mRNA splicing to generate XBP1s, and also mediates regulated IRE1‐dependent decay (RIDD) of target mRNAs. PERK phosphorylates eIF2α, resulting in global translational attenuation and selective translation of ATF4, which induces genes involved in redox control, amino acid metabolism, and CHOP‐mediated apoptotic signaling. ATF6 translocates to the Golgi apparatus, where sequential cleavage by S1P and S2P generates the active transcription factor ATF6‐N. The three UPR branches coordinate to restore ER proteostasis through transcriptional upregulation of chaperones, folding enzymes, and ER‐associated degradation components. Crosstalk among the UPR sensors modulates the amplitude and duration of stress signaling, the PERK‐ATF4‐ATF6 regulatory loop and XBP1s‐ATF6 transcriptional cooperation. Mitochondria‐associated ER membranes (MAMs) contribute to UPR regulation through Ca^2+^ exchange and ROS production, influencing sensor activation and apoptotic output. Under conditions of prolonged or unresolvable ER stress, convergence of UPR signaling pathways shifts the cellular response toward apoptosis through Bcl‐2 family proteins and JNK‐dependent mechanisms.

The accumulation of excess unfolded or misfolded proteins in the ER triggers activation of the UPR. These aberrant proteins can bind to BiP or directly engage three transmembrane sensors located on the ER membrane: inositol requiring enzyme 1α (IRE1α), pancreatic ER kinase‐like ER kinase (PERK), and activating transcription factor 6 (ATF6) (both α and β isoforms) [9]. Activation of these sensors initiates three major UPR signaling pathways—IRE1α‐XBP1, PERK‐eIF2α‐ATF4, and ATF6—which work in concert to restore ER homeostasis [10, 11, 12]. However, under conditions of prolonged or unresolvable ER stress, these initially cytoprotective pathways undergo a decisive shift toward pro‐apoptotic signaling, culminating in programmed cell death. During this process, the host UPR also regulates autophagy and apoptotic mechanisms to mitigate damage and maintain cellular integrity (Figure 1) [13].

### 2.2 IRE1α‐XBP1 Pathway Manipulation

IRE1α is a type I transmembrane protein residing in the ER with nuclease activity that possesses endoribonuclease activity and plays a key role in transmitting stress signals from the ER to other organelles, including the mitochondria [5]. During ER stress conditions, IRE1 dissociates from the chaperone Bip, and its kinase domain dimerizes and undergoes autophosphorylation, which in turn activates its RNase function [14]. This endoribonuclease activity cleaves the substrate X‐box binding protein 1 (*XBP1*) mRNA, generating *XBP1s* (X‐box binding protein 1 splicing) mRNA [15]. The resulting XBP1s transcription factor orchestrates the expression of genes governing protein folding machinery, ERAD pathways, and lipid metabolism. It also promotes ER lipid synthesis, peptide folding, and membrane expansion, thereby facilitating cellular adaptation to ER stress [16, 17, 18, 19].

Recent studies have revealed that XBP1s regulates a broader repertoire of target genes beyond its classical ER stress‐responsive transcriptome, including pro‐inflammatory cytokines, cell differentiation programs, and the hexosamine biosynthetic pathway [20]. The XBP1s transcription factor exhibits strong regulatory associations with genes encoding cell adhesion molecules and cytokine‐cytokine receptor interactions, thereby modulating immune system responses and inflammatory processes [21]. Subsequent investigations by Dong et al. demonstrated that XBP1s enhances oxidative phosphorylation in natural killer (NK) cells [22]. Additionally, these researchers identified c‐Myc as a downstream transcriptional target of XBP1s that governs NK cell proliferation. This IRE1α‐XBP1s‐c‐Myc signaling pathway orchestrates NK cell effector functions, thereby influencing both cellular immunity and anti‐tumor immune responses [23].

### 2.3 PERK‐eIF2α‐ATF4 Translational Control

Following dissociation from Bip, PERK undergoes dimerization or oligomerization which induces a conformational change to expose its kinase domain. This activation leads to the phosphorylation of eukaryotic initiation factor 2α (eIF2α), thereby inhibiting the synthesis of secretory proteins [24], while electively promoting the translation of activating transcription factor 4 (ATF4). The transcription factor ATF4, which is transiently upregulated during the acute phase of ER stress, coordinates the transcriptional activation of genes involved in amino acid metabolism and redox homeostasis [25]. Subsequently, ATF4 induces downstream effectors including C/EBP homologous protein (CHOP) and growth arrest/DNA damage‐inducible protein 34 (GADD34), which further contribute to the adaptive response [24]. However, under persistent ER stress, the CHOP‐mediated apoptotic pathway is activated to eliminate damaged cells [26].

### 2.4 ATF6 Transcriptional Pathway

ATF6 represents a group of ER stress transducers that encode basic Leu zipper (bZIP) transcription factors, including ATF6α, ATF6β, LUMAN (also known as CREB3), old astrocyte specifically‐induced substance (OASIS; also known as CREB3L1), BBF2 human homologue on chromosome 7 (BBF2H7; also known as CREB3L2), cyclic AMP‐responsive element‐binding protein hepatocyte (CREBH; also known as CREB3L3), and CREB4 (also known as CREB3L4) [27]. During ER stress, ATF6 traffics from the ER membrane to the Golgi apparatus via the conventional secretory pathway. Within the Golgi compartments, ATF6 undergoes sequential proteolytic cleavage by site‐1 protease (S1P) and site‐2 metalloprotease (S2P), which releases its cytosolic bZIP transcription factor domain (ATF6‐N) [28]. The liberated ATF6‐N subsequently translocates to the nucleus, where it transcriptionally upregulates genes encoding ER chaperones and folding machinery, including calreticulin and calnexin, thereby facilitating the ER's adaptive response to restore ER homeostasis [16].

### 2.5 Regulation of the UPR by Mitochondria

In addition to the signal crosstalk between UPR sensors, mitochondria also play a decisive role in the response direction of the UPR, and serve as the core hub for maintaining the ER‐mitochondrial stress balance [29]. Mitochondria regulate UPR signal transmission through MAMs (mitochondria‐associated ER membranes). As the physical connection site between the two, the integrity of MAMs is synergistically maintained by Mfn‐2 (mitofusin 2) and UPR sensors (such as PERK). Deficiency of Mfn‐2 leads to MAMs disruption and excessive activation of the UPR; meanwhile, MAMs‐mediated Ca^2+^ homeostasis transport directly affects the level of ER stress, thereby regulating the activation intensity of the UPR [30]. Mitochondrial dysfunction leads to excessive accumulation of ROS (reactive oxygen species). ROS can oxidize UPR sensors (such as IRE1α and PERK) to regulate signal output, and also disrupt the ER protein folding environment, indirectly amplifying the UPR [31]. Mitochondria‐related molecules such as Siah1/2 can be upregulated by UPR transducers. They enhance ATF4 activity by stabilizing HIF1α to regulate UPR signal output, and can also indirectly affect the UPR apoptotic‐oriented response by regulating the mitochondrial fission complex. Bacteria can secrete effector proteins to interfere with mitochondrial function, disrupt MAMs integrity, or regulate, thereby directionally remodeling the UPR response to support their own intracellular survival [32].

## 3 Bacterial Strategies for ER Exploitation

### 3.1 Establishing ER‐Associated Replicative Niches

Viral infections have been extensively documented to disrupt ER homeostasis through the massive synthesis, modification, and processing of viral proteins [33]. To facilitate their replication and proliferation, viruses strategically hijack the host UPR machinery, establishing a favorable intracellular milieu that promotes viral persistence while facilitating evasion of host immune surveillance [8, 34]. Similarly, bacterial pathogens have evolved sophisticated strategies to manipulate host UPR signaling pathways for survival and replication. Many bacteria establish specialized replication niches within host‐derived membranous compartments known as bacteria‐containing vacuoles (BCVs). These membrane contact sites facilitate the bidirectional exchange of lipids, proteins, and signaling molecules between pathogens and hosts, playing essential roles in pathogen survival.

*Brucella* species initially allow their bacteria‐containing vacuoles (BCVs) to mature along the endocytic pathway, subsequently establishing sustained interactions with ER exit sites (ERES) through VirB T4SS‐dependent mechanisms. This process progressively replaces endocytic membranes with ER‐derived membranes, generating replication‐competent BCVs [35].

*Legionella pneumophila* establishes a *Legionella*‐containing vacuole (LCV) that circumvents endosomal maturation by intercepting early secretory vesicles trafficking between the ER and Golgi apparatus. Through type IV secretion system (T4SS) effector proteins, *L. pneumophila* coordinately manipulates host trafficking machinery, ultimately fusing with ER membranes to form a replication‐permissive organelle [36].

Although these strategies employ distinct pathways, they converge on a common mechanism: precise manipulation of host secretory systems through effector proteins and metabolic perturbations to establish favorable replication environments. The following sections detail key effector proteins and host factors co‐opted in these processes.

### 3.2 Bacterial Pathogen‐Induced Activation of Host UPR Signaling

Certain intracellular bacterial pathogens activate host UPR signaling through virulence toxin or effector protein secretion, enhancing survival and utilizing host ER to establish protective niches and secure nutritional resources (Table 1).

**TABLE 1:** Bacterial toxins and effector proteins that Induces the unfolded protein response.

Bacteria	Toxins/Effector	Mode of action	References
*Vibro cholerae*	CT	CTxA1 binds to IRE1αLD pocket through local unfolding of I124‐F132 motif, triggering IRE1α dimerization/oligomerization	[37]
*Brucella*	VceC	Localizes to the endoplasmic reticulum, where it interacts with the luminal chaperone BiP/GRP78	[38]
	BspB	Targets COG complex (binds COG6); forms regulatory network with RicA and Rab2 to redirect Golgi‐derived vesicles	[39]
	TcpB	Upregulates UPR target gene expression and enhances *XBP1* mRNA splicing during infection	[40]
	BspL	Interacts with the central component of ERAD machinery HERP	[41]
*M. tuberculosis*	Rv0297	Disruption of Ca^2+^ homeostasis and increased production of nitric oxide (NO)	[42]
	ESAT‐6 HBHA	Increases intracellular Ca^2+^ and Reactive Oxygen Species (ROS)	[43]
	CdhM	Localizes to ER; induces ER morphological abnormalities	[44]
*Legionella*	Lpg0519	Localizes to ER; directly activates atypical ATF6 pathway	[45]
*Listeria*	LLO	Cholesterol‐dependent cytolysin; binds host cell membrane and forms membrane pores; disrupts Ca^2+^ homeostasis	[46]

*Vibrio cholerae* releases cholera toxin (CT), which then enters cells via receptor‐mediated endocytosis after binding to the GM1 ganglioside receptor and undergoes retrograde transport through the Golgi apparatus to the ER. Within the ER lumen, the disulfide bond linking the A1 and A2 subunits of CT is cleaved through reduction mediated by protein disulfide isomerase, releasing the enzymatically active A1 subunit (CTxA1). Upon entering the host cell's ER, CTxA1 does not require global unfolding; instead, only local unfolding of a seven‐residue motif (I124‐F132) on its edge β‐strand is necessary for it to bind to a specific pocket on the IRE1α luminal domain (IRE1αLD). This pocket is normally occupied by the Y358‐P368 fragment of IRE1α's own C‐terminal flexible loop, and CTxA1 competes for binding by mimicking the structural characteristics of this fragment. Notably, the B subunit of CT (CTxB) lacks this binding activity. This binding event triggers the formation of IRE1α dimers or small oligomers, activating its kinase‐endonuclease activity [47]. Subsequently, IRE1α splices *XBP1* mRNA to generate the transcription factor XBP1s, which initiates the expression of downstream genes of the UPR (e.g., ERdj4). Ultimately, this process enhances CT‐induced cyclic adenosine monophosphate (cAMP)‐dependent fluid secretion, exacerbating the diarrheal symptoms of cholera [37].

*Brucella* employs multiple effector proteins to manipulate host UPR signaling: T4SS effector VceC exemplifies this strategy (Table 1). VceC localizes to the ER membrane via its N‐terminal transmembrane domain and specifically binds to BiP, the major molecular chaperone of the ER, in the ER lumen. Under normal conditions, BiP binds to the ER stress sensor IRE1α to maintain its inactive state, while the binding of VceC to BiP induces the dissociation of BiP from IRE1α, thereby triggering the phosphorylation and oligomerization of IRE1α and ultimately activating the IRE1 pathway. The activated IRE1α specifically degrades the host's *Bloc1s1* mRNA through its Regulated IRE1‐Dependent Decay (RIDD) function, leading to a reduction in BLOS1 protein and impairment of the BLOC‐1 complex, which normally mediates the fusion of lysosomes with endosomes. This prevents the Brucella‐containing vacuole (BCV) from fusing with lysosomes, allowing *Brucella* to evade degradation by lysosomal enzymes. Meanwhile, the ER membrane expansion and remodeling induced by UPR activation helps the replicative BCV (rBCV) acquire ER membrane components, facilitating the formation of a favorable intracellular niche for *Brucella* replication [48]; Another effector, TcpB, significantly upregulates the expression of UPR target genes including BiP, CHOP, and ERdj, while enhancing *XBP1* mRNA splicing during *Brucella* infection (Table 1) [40]; In addition, activated IRE1α can recruit TRAF2 through MAMs, thereby activating the JNK signaling pathway on mitochondria, promoting mitochondrial membrane potential depolarization, and releasing cytochrome c [49]; however, XBP1s simultaneously upregulates the expression of the mitochondrial anti‐apoptotic protein Bcl‐2 to balance apoptotic signals, ultimately maintaining host cell survival to support bacterial replication.

BspB does not directly initiate the UPR but instead targets the Conserved Oligomeric Golgi (COG) complex on the host's Golgi apparatus (binding to subunits such as COG6), disrupting the normal retrograde transport of the Golgi. Meanwhile, it forms a regulatory network with the effector protein RicA and the host small GTPase Rab2, redirecting vesicles containing membrane lipids and proteins that would otherwise cycle within the Golgi or be recycled back to the ER. By hijacking these “newly synthesized membrane materials” derived from the ER and transported via the Golgi apparatus, BspB delivers them to the *Brucella* replicative vacuole (rBCV), providing membrane sources for the rBCV, facilitating its camouflage and maturation, and ultimately enabling the formation of an intracellular niche suitable for bacterial proliferation [39, 50].

*Mycobacterium tuberculosis* employs both effector proteins and antigenic molecules to manipulate host ER function [51, 52]. When cells are infected with *Mycobacterium tuberculosis* , the bacterium secretes effector proteins such as CdhM. The effector protein CdhM localizes to the ER, where it induces morphological abnormalities and activates both the IRE1α and PERK pathways of the UPR (Table 1). This UPR activation leads to macrophage apoptosis, facilitating bacterial survival and replication within host cells [44]. These effector proteins not only directly regulate ER function but also enhance bacterial survival during infection by interfering with MCSs. Meanwhile, ER calcium efflux induced by HBHA can activate lysosomal calcium signaling through MCSs, promoting the separation of lysosomes from BCVs (bacterial‐containing vacuoles) to avoid bacterial degradation by lysosomes, thereby indirectly reinforcing the pro‐survival signals of the UPR [43, 53].

*Legionella* is another intracellular bacterial pathogen that exploits the ER for replication. Its effector protein Lpg0519 localizes to the ER and directly activates the atypical pathway of ATF6 to induce the UPR (Table 1) [45]. LLO of *Listeria monocytogenes* is a cholesterol‐dependent cytolysin (Table 1). After secretion, it binds to the host cell membrane and assembles into membrane pores, disrupting the host cell's Ca^2+^ homeostasis to trigger ER stress. This leads to the accumulation of unfolded proteins, which in turn causes the ER‐resident chaperone BiP to dissociate from the three UPR sensors (PERK, IRE1, and ATF6), thereby initiating pathway activation [46].

In summary, bacterial infections induce ER stress and modulate UPR pathways through diverse mechanisms to create an intracellular environment that favors bacterial replication (Table 1). These strategies include direct interaction with ER chaperones, disruption of ER‐Golgi trafficking, calcium homeostasis perturbation, and selective activation of specific UPR pathways.

### 3.3 Bacterial Pathogens Inhibit UPR Signaling

**FIGURE 2:**
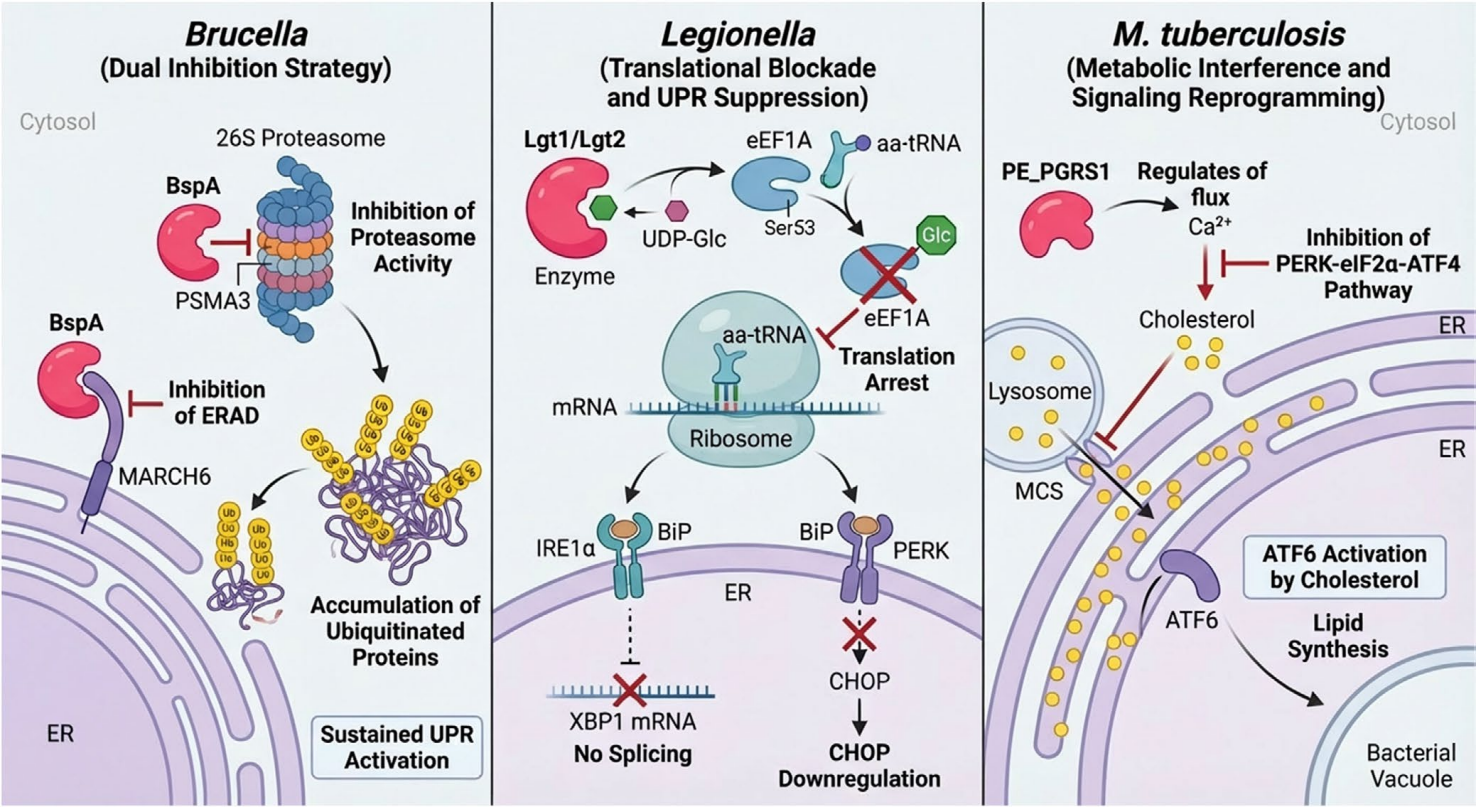
Bacterial inhibition of the unfolded protein response (UPR) during infection. (Left) *Brucella* effector BspA inhibits ERAD by targeting the E3 ligase MARCH6 and blocks proteasome activity via PSMA3, sustaining UPR activation to protect bacterial effectors. (Middle) *Legionella* effectors Lgt1/2 glucosylate host eEF1A, arresting translation and preventing the induction of IRE1α and PERK pathways. (Right) *M. tuberculosis* effector PE_PGRS1 alters host Ca^2+^ flux to inhibit the PERK pathway, while impaired lysosomal transport leads to cholesterol accumulation and ATF6 activation, supporting vacuole membrane lipid synthesis.

While UPR activation provides bacteria with replication advantages, the same pathways can threaten bacterial survival through pro‐apoptotic and inflammatory responses. Consequently, pathogens have evolved complementary strategies to selectively suppress detrimental UPR outputs while maintaining beneficial ones (Figure 2). During bacterial infections, the UPR initially serves as a cellular defense mechanism. Pathogens employ sophisticated mechanisms to directly suppress host UPR signaling pathways and compromise the function of associated proteins, thereby creating a more favorable environment for their replication and survival. These strategies demonstrate the evolutionary adaptation of bacterial pathogens to exploit host cellular machinery while simultaneously evading defensive responses.

The *Brucella* effector protein BspA inhibits ER‐associated degradation (ERAD) by targeting the host E3 ubiquitin ligase MARCH6, effectively reducing stress signal transduction. It can also bind to the host proteasome subunit PSMA3 to inhibit the peptidase activity of the proteasome. This “dual inhibition of ERAD^+^ proteasome” strategy prevents the degradation of ubiquitinated misfolded proteins, continuously activates the pro‐survival branches of the UPR, and simultaneously avoids the degradation of its own effector proteins by the UPS, thereby enhancing bacterial pathogenicity [54].

The effector proteins Lgt1 and Lgt2 of *Legionella pneumophila* inhibit the UPR through a unique mechanism (Figure 2). They do not directly bind to UPR sensors but rather act as GT‐A family glucosyltransferases. Using UDP‐glucose within host cells as a donor, these proteins catalyze mono‐O‐glucosylation at the serine‐53 (Ser53) residue of the host's eukaryotic translation elongation factor 1A (eEF1A). This modification alters the spatial conformation of eEF1A; while it does not affect eEF1A's ability to bind GTP, it significantly reduces its capacity to bind aminoacyl‐tRNA and impedes its interaction with ribosomes, leading to the arrest of translation elongation and the cessation of overall protein synthesis in host cells [55]. Consequently, newly synthesized polypeptide chains cannot enter the ER, which reduces the protein load in the ER lumen and allows existing unfolded proteins to be processed completely, thereby eliminating ER stress. This process specifically inhibits the inositol‐requiring kinase 1α (IRE1α) branch of the UPR—blocking the splicing of *XBP1* mRNA and the production of XBP1s protein induced by chemicals (such as thapsigargin and tunicamycin or pathogen‐associated molecular patterns (PAMPs)), suppressing the transcription of XBP1s downstream target genes (e.g., ERdj4), and attenuating the linkage of proinflammatory signals, as well as inhibiting the PKR‐like ER kinase (PERK) pathway (e.g., C/EBP homologous protein [CHOP] expression). Ultimately, this fundamentally eliminates the trigger for ER stress [56].

The *Mycobacterium tuberculosis* effector protein PE_PGRS1 manipulates host cellular homeostasis by regulating intracellular Ca^2+^ levels, which subsequently inhibits the PERK‐eIF2α‐ATF4 signaling pathway (Figure 2). ATF4 upregulates the expression of amino acid synthesis genes, which, while alleviating ER stress, also provides essential nutrients for the bacteria; meanwhile, impaired lysosomal cholesterol transport causes cholesterol accumulation in the ER membrane, activating the ATF6 pathway and upregulating lipid synthesis genes to support the membrane structure of BCVs (bacterial‐containing vacuoles). This mechanism of “lysosome‐ER crosstalk disorder → directed activation of UPR” is one of the key strategies for the long‐term intracellular survival of *Mycobacterium tuberculosis* [42].

## 4 ER‐Phagy Manipulation by Bacterial Pathogens

In addition to the classical autophagy induction pathways, ERS has been recognized as one of the key regulators of cellular autophagy [57]. ER stress serves as a key autophagy regulator in maintaining cellular homeostasis. ER stress‐mediated autophagy and the proteasomal degradation system (ubiquitin‐proteasome system, UPS) exhibit close synergy: unfolded proteins are first ubiquitinated and labeled through the ERAD pathway; if ERAD fails to achieve complete clearance, ER autophagy is initiated to degrade damaged ER fragments [58]. Bacterial pathogens can target both systems simultaneously: on the one hand, they reduce the degradation of their own effector proteins by inhibiting proteasomal activity; on the other hand, they regulate the autophagic pathway to obtain nutrients or evade immunity. Under physiological conditions, specific ER autophagy receptor proteins including FAM134B, RTN3, SEC62, and CCPG1 recognize protein aggregates and eliminate damaged protein regions and misfolded proteins, thereby restoring ER function [59, 60].

In addition to the classical autophagy induction pathways, ERS has been recognized as one of the key regulators of cellular autophagy [57]. Increasing evidence suggests that ERS‐mediated autophagy plays a critical role in restoring ER function and maintaining cellular homeostasis [11, 61]. When unfolded or misfolded proteins accumulate in the ER, ERS is triggered. If the stress response cannot be alleviated, ER‐associated autophagy is activated. Specific ER autophagy receptor proteins, such as FAM134B, RTN3, SEC62, and CCPG1, can recognize protein aggregates and eliminate damaged protein regions and misfolded proteins [59, 60].

**FIGURE 3:**
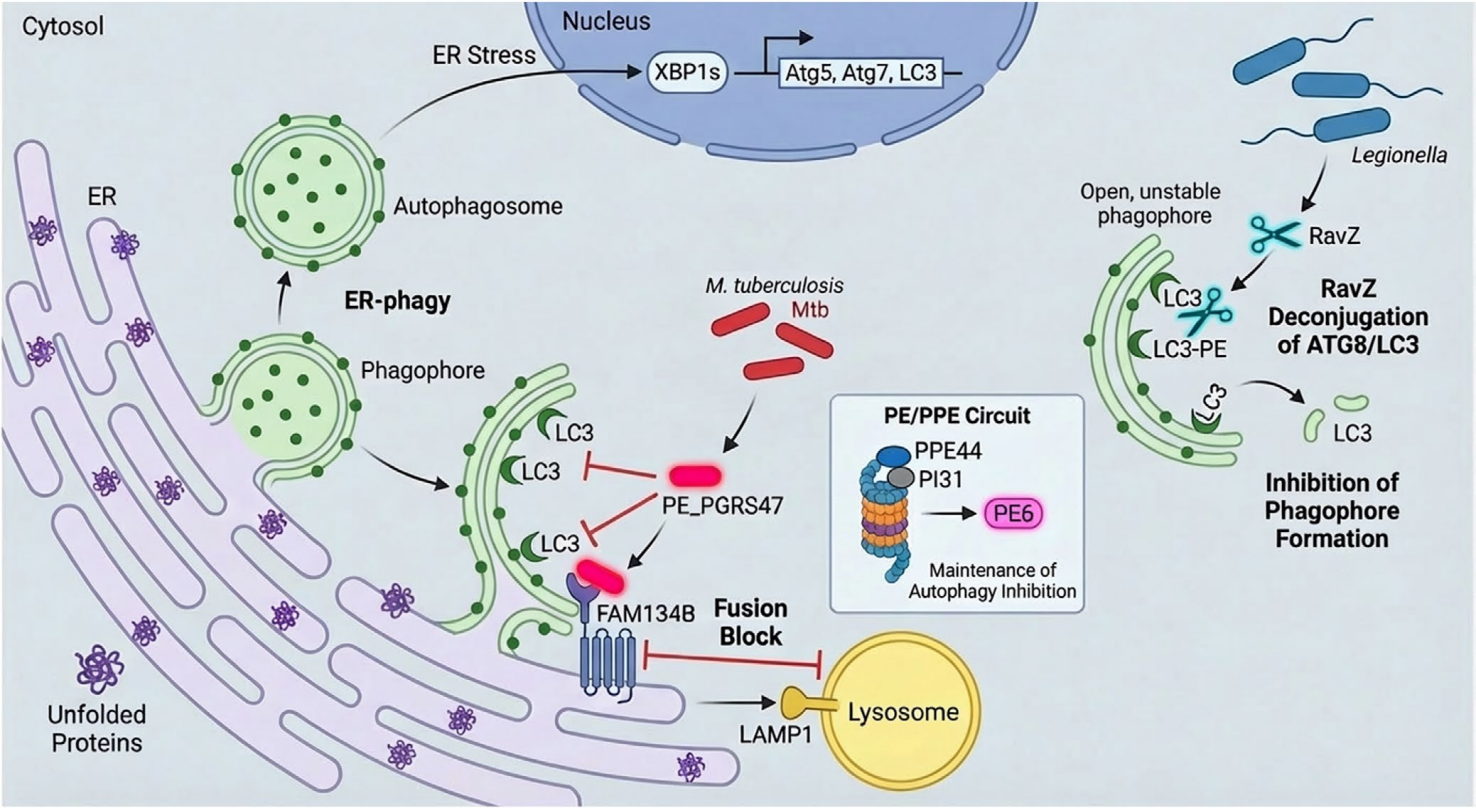
Bacterial modulation of ER‐phagy and host autophagy pathways during intracellular infection. *Mycobacterium tuberculosis* (Mtb) infection triggers ER stress, leading to XBP1s‐mediated upregulation of autophagy genes (*Atg5*, *Atg7*, *LC3*) and the formation of ER‐derived phagophores. To counteract this, *Mtb* deploys specific effectors: (1) The PE/PPE circuit, where PE6 inhibits mTOR signaling but is regulated by the proteasome; the effector PPE44 binds the proteasome regulator PI31 to enhance proteasomal activity and modulate PE6 stability. (2) PE_PGRS47 binds the ER‐phagy receptor FAM134B, blocking ER‐derived autophagosome formation and inhibiting FAM134B‐LAMP1 interaction to prevent lysosomal fusion. In *Legionella pneumophila* infection (right), the effector RavZ targets host autophagy by irreversibly deconjugating ATG8/LC3 from autophagosomal membranes. This cleavage prevents phagophore maturation and functional autophagosome formation, disrupting the host's clearance mechanism.

Bacteria have evolved sophisticated mechanisms to hijack host cell autophagy for survival and proliferation. Different bacterial pathogens employ distinct strategies to manipulate ER‐phagy: *Mycobacterium tuberculosis* (Mtb) infection triggers ER stress and activates the UPR (Figure 3). XBP1s directly transcriptionally activates autophagy‐related genes (*Atg5*, *Atg*7, *LC3*); the ER membrane forms LC3‐containing phagophores through “budding”, which encapsulate intracellular Mtb (or the phagosomes containing Mtb) to form autophagosomes—studies have confirmed that approximately 30% of autophagosomal membranes are directly derived from the ER [62]. *Mycobacterium tuberculosis* secretes PE/PPE proteins that play crucial roles in modulating host cell death pathways. The PE6 protein inhibits autophagy by suppressing the MTOR signal; meanwhile, its ubiquitination modification can be recognized and degraded by the host proteasome. In contrast, the PPE44 secreted by the bacterium can bind to the proteasome inhibitor PI31, enhance proteasomal activity, accelerate PE6 degradation, and maintain the balance of autophagy inhibition [63]. PE_PGRS47 can specifically bind to the autophagic domain of the ER autophagy receptor FAM134B; it not only inhibits the formation of ER‐derived autophagosomes but also reduces the interaction between FAM134B and the lysosomal membrane protein LAMP1, directly impairing the fusion efficiency between ER‐derived autophagosomes and lysosomes [64]. Other members of the PE/PPE family, including PE_PGRS20, PE_PGRS21, PE_PGRS30, PE_PGRS47, PPE44, and PPE51, also exhibit autophagy inhibitory functions [65]. These findings demonstrate that the PE/PPE family proteins of *M. tuberculosis* inhibit host autophagy and immune responses, thereby enhancing bacterial intracellular survival, virulence, and pathogenicity [66].

*Legionella pneumophila* utilizes ER‐phagy to acquire nutrients (Figure 3). The effector protein RavZ irreversibly deconjugates the ATG8 protein from the autophagosomal membrane, thereby inhibiting the formation of conventional ATG8^+^ autophagosomes [67], thereby inhibiting the formation of conventional ATG8^+^ autophagosomes and compromising autophagy in infected cells [68].

## 5 Pathogen‐Mediated Immune Response Regulation

**FIGURE 4:**
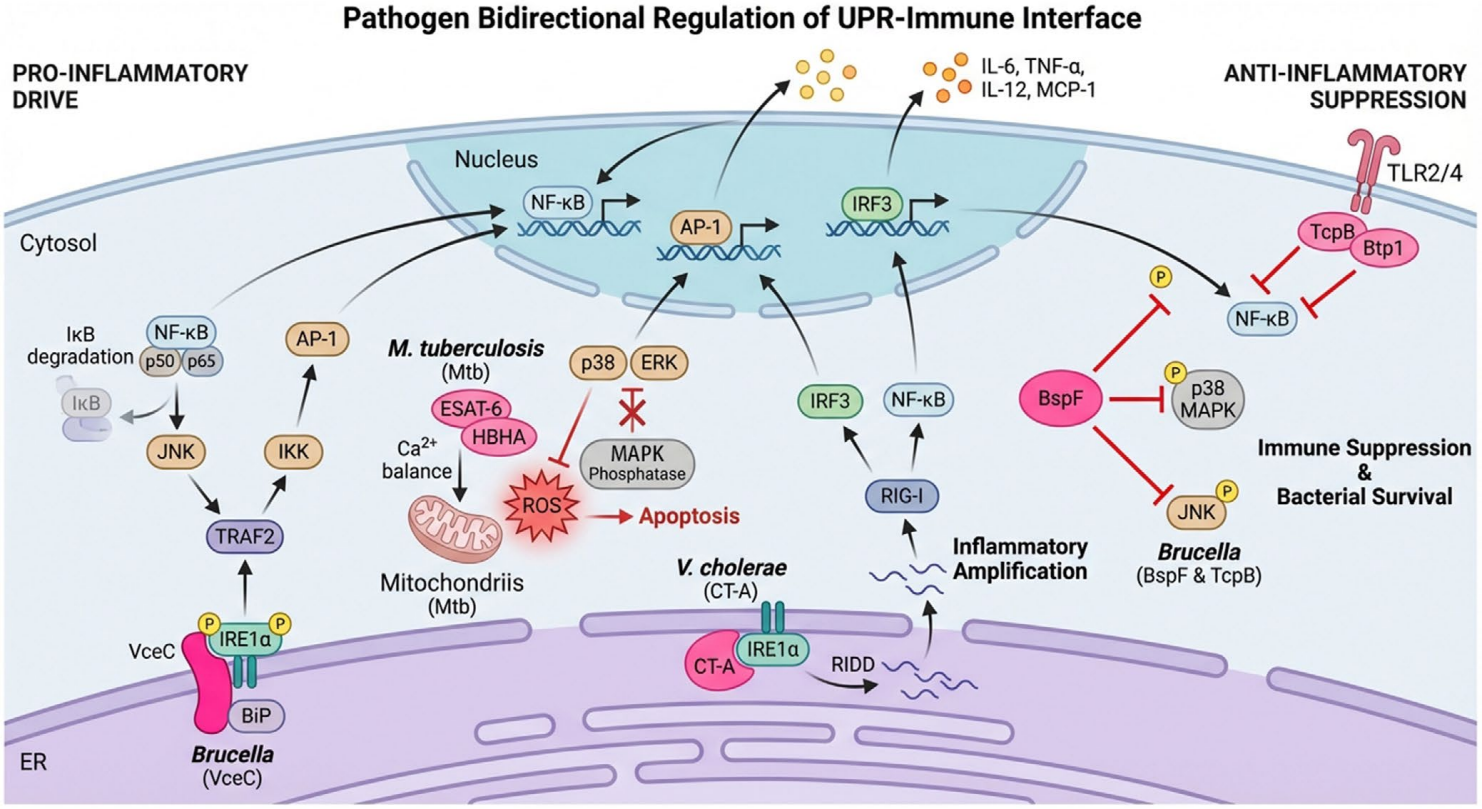
Pathogen‐mediated bidirectional regulation of the unfolded protein response–immune interface. Bacterial pathogens modulate inflammatory outputs through UPR sensors. Pro‐inflammatory drive: Brucella VceC activates IRE1α‐TRAF2‐JNK signaling; *M. tuberculosis* effectors (ESAT‐6, HBHA) induce ROS and sustain MAPK activation; *V. cholerae* CT‐A triggers IRE1α‐dependent RIDD and NF‐κB signaling. Anti‐inflammatory suppression: Brucella effectors BspF and TcpB/Btp1 inhibit NF‐κB and MAPK pathways to dampen cytokine secretion and promote bacterial persistence.

UPR signaling plays pivotal roles in shaping host immune responses during infection. *Brucella* T4SS effector protein VceC interacts with BiP, activating the IRE1‐XBP1 pathway (Figure 4). Phosphorylated IRE1 recruits TNFα receptor‐associated factor 2 (TRAF2), activating inhibitor of NF‐κB (IκB) kinase and c‐Jun N‐terminal kinase (JNK) signaling cascades, leading to IκB phosphorylation and degradation. Ultimately, this activates transcription factors NF‐κB and AP‐1, resulting in pro‐inflammatory cytokine secretion including IL‐6 and TNF‐α [38].

*Mycobacterium tuberculosis* effector proteins ESAT‐6 and HBHA promote reactive oxygen species (ROS) production by disrupting intracellular calcium homeostasis (Figure 4). During Mycobacterium infection, HBHA stimulation induces ROS accumulation within infected cells. Resulting oxidative stress inactivates mitogen‐activated protein kinase (MAPK) phosphatases through catalytic cysteine residues, driving sustained MAPK activation and upregulating pro‐inflammatory cytokine production including IL‐6 and monocyte chemoattractant protein‐1 (MCP‐1). This inflammatory cascade culminates in ER stress‐mediated apoptosis, with oxidative stress activating the NF‐κB/JNK pathway and MAPK signaling, leading to pro‐inflammatory cytokine production including TNF‐α, IL‐6, IL‐12, and MCP‐1 (Figure 3) [69]. Additionally, the folded cholera toxin A subunit directly binds to IRE1α, inducing regulated IRE1‐dependent decay (RIDD) and activating the NF‐κB pathway (Figure 4). This activation occurs through engagement of the RIG‐I‐mediated single‐stranded RNA innate immune sensing pathway, creating an additional layer of inflammatory response amplification (Figure 3) [37].

Conversely, pathogens counteract UPR to suppress host immune responses, weakening the host's ability to clear infections and creating complex immunoregulatory balance. *Brucella* T4SS effector protein BspF inhibits NF‐κB, p38 MAPK, and JNK MAPK signaling pathway activation, suppressing pro‐inflammatory cytokine secretion including IL‐6 and TNF‐α [70]. Effector protein Btp1/TcpB inhibits Toll‐like receptor 2 (TLR2) and TLR4‐mediated NF‐κB activation, further dampening host immune responses while promoting *Brucella* survival and intracellular replication [40, 71].

## 6 Therapeutic Targeting of Endoplasmic Reticulum Stress Pathways

The pivotal role of ER stress in diverse diseases has spurred development of therapeutic strategies targeting either UPR signaling pathways or direct ER environment modulation. These approaches show promise not only in cancer and metabolic diseases but also in disrupting pathogen‐induced ER stress for antimicrobial applications.

### 6.1 PERK Pathway Inhibitors

PERK inhibitors such as GSK2606414 and AMG44 have shown promise in preclinical cancer studies by preventing eIF2α phosphorylation and maintaining protein synthesis under stress conditions [72, 73, 74].

In bacterial infections, PERK inhibition may serve dual therapeutic purposes by preventing pathogen‐induced translational shutdown that impairs host immune protein synthesis and by disrupting bacterial exploitation of PERK signaling pathways to establish replication niches. For instance, treatment with the PERK inhibitor GSK2656157 in *Mycobacterium tuberculosis* infection effectively attenuated pyroptosis while simultaneously reducing bacterial burden and ameliorating pathological damage in lung tissues [75]. However, PERK inhibition carries significant risks including pancreatic β‐cell toxicity and potential exacerbation of ER stress in healthy tissues [75]. The therapeutic window for infectious disease applications would require careful dose optimization and potentially tissue‐specific delivery approaches [76]. Combination strategies using PERK inhibitors alongside traditional antimicrobials could provide synergistic effects while minimizing individual drug toxicities. Targeting downstream PERK effectors, the small molecule integrated stress response inhibitor (ISRIB) selectively antagonizes phosphorylated eIF2α binding to eIF2B, stabilizing the guanine nucleotide exchange factor's active conformation and restoring global protein synthesis despite ongoing ER stress [77].

### 6.2 IRE1α Pathway Inhibitors

IRE1α represents a particularly attractive target due to its dual enzymatic activities, allowing for selective intervention strategies. Kinase domain inhibitors such as 4μ8c and KIRA6 prevent IRE1α autophosphorylation and downstream JNK/NF‐κB activation without affecting XBP1 splicing [78]. This selective inhibition could be valuable for infections where pathogen‐induced inflammation is more harmful than beneficial. Conversely, RNase‐specific inhibitors such as STF‐083010 and 3,5‐dibromosalicylaldehyde target XBP1 splicing while preserving kinase activity. This approach might be beneficial for infections where inflammatory signaling helps host defense but pathogen‐induced XBP1s promotes bacterial survival [79]. The development of next‐generation IRE1α modulators with improved selectivity and pharmacological properties represents an active area of therapeutic development [80]. MKC‐3946 is a selective inhibitor of IRE1α that suppresses XBP1 splicing and activation and has demonstrated therapeutic potential in multiple myeloma and solid tumors [81, 82].

### 6.3 ATF6 Pathway Inhibitors

ATF6 pathway modulation presents unique opportunities for infectious disease intervention. ATF6 activators such as Compound 147 could enhance host ER folding capacity and stress resistance, potentially improving immune cell function during infection [83]. Alternatively, ATF6 inhibitors like Ceapins could disrupt pathogen‐induced ATF6 signaling that promotes infection establishment. Ceapin compounds represent the first selective ATF6 inhibitors, with Ceapin‐A7 showing potent activity through stabilization of ATF6 oligomeric complexes within the ER lumen [84]. The generally cytoprotective nature of ATF6 signaling suggests that activation strategies may be more broadly applicable than inhibition approaches. However, the optimal strategy would depend on the specific pathogen and infection context, requiring a detailed understanding of how individual bacterial species exploit ATF6 signaling [85].

### 6.4 Direct ER Environment Modulators

In contrast to UPR‐modulating agents, compounds like thapsigargin represent a strategy of direct ER perturbation. Thapsigargin (TG) functions as a highly selective and irreversible inhibitor of the ER Ca^2+^‐ATPase (SERCA) pump [86]. This potent cytotoxin disrupts cellular calcium homeostasis by blocking Ca^2+^ transport from the cytoplasm into the ER. This results in elevated cytosolic Ca^2+^ levels and depleted ER Ca^2+^ stores [87, 88]. This dual perturbation induces severe ER stress, promotes the accumulation of misfolded proteins, and ultimately triggers apoptotic cell death [89].

### 6.5 Challenges in Therapeutic Development and Future Directions

A fundamental challenge in developing UPR‐targeted therapeutics for infectious diseases lies in achieving selectivity for infected versus uninfected cells. Unlike cancer applications where tumor‐specific targeting mechanisms can be employed, bacterial infections typically involve normal host cells harboring pathogens, making selective intervention considerably more complex. Nonspecific UPR modulation poses significant risks to healthy tissues, particularly those with high secretory demands such as pancreatic β‐cells, plasma cells, and hepatocytes [90, 91].

Several strategic approaches have emerged to address this selectivity challenge. The development of pathogen‐responsive drug delivery systems represents a promising avenue; wherein therapeutic activation occurs specifically within infected tissue environments. Alternative approaches focus on targeting pathogen‐specific UPR manipulation mechanisms rather than broad‐spectrum UPR signaling pathways. Combination therapy strategies offer the potential to minimize individual drug doses while maintaining therapeutic efficacy, thereby reducing systemic toxicity. Additionally, temporal administration strategies that align with infection kinetics and host response patterns may enhance therapeutic windows while minimizing adverse effects [91, 92].

The potential for bacterial resistance to UPR‐targeted therapeutics presents another significant developmental challenge. Pathogens may evolve resistance through acquisition of alternative UPR manipulation strategies, enhanced resistance to ER stress via improved chaperone expression or alternative protein folding pathways, metabolic adaptations that reduce dependence on UPR manipulation, and temporal shifts in effector expression that circumvent inhibited pathways. Understanding these resistance mechanisms is crucial for rational therapeutic design. Combination strategies targeting multiple UPR branches or integrating UPR modulators with conventional antimicrobials could substantially reduce resistance development potential [76]. Furthermore, targeting host‐pathogen interaction interfaces rather than essential bacterial functions may provide higher evolutionary barriers to resistance development [92].

The therapeutic landscape for UPR‐targeted infectious disease interventions continues evolving rapidly, with several key areas emerging for future development [85]. Pathogen‐specific UPR modulators designed based on detailed mechanistic understanding of individual bacterial manipulation strategies represent a critical research priority [4]. Host‐directed therapies that enhance beneficial UPR responses while preventing pathogen exploitation offer complementary therapeutic approaches. Integration of UPR modulation with traditional antimicrobials and immunomodulators through sophisticated combination strategies may yield synergistic therapeutic benefits [92]. Precision medicine approaches that tailor UPR interventions to specific patient populations and infection contexts represent an emerging paradigm for optimizing therapeutic outcome [76].

Technological advances in drug delivery systems, including sophisticated nanoparticle formulations and tissue‐specific targeting approaches, will prove essential for achieving therapeutic efficacy while minimizing off target effects. The integration of biomarker discovery with therapeutic development programs could enable personalized treatment strategies based on individual patient UPR status and specific pathogen characteristics, ultimately advancing the field toward more precise and effective interventions [85].

## 7 Summary

The ER represents a critical battleground where intracellular bacterial infection outcomes are determined through complex molecular negotiations. Pathogens including *Brucella*, *Mycobacterium tuberculosis* , *Legionella*, and *Salmonella* have evolved sophisticated mechanisms to establish membrane contact sites, manipulate UPR signaling, and subvert ER‐phagy, transforming host stress responses into survival advantages.

The UPR demonstrates remarkable complexity during bacterial infection, functioning both as a host defense mechanism and a pathogen exploitation target. While UPR activation initiates protective responses such as autophagy, inflammation, and apoptosis to eliminate pathogens, it simultaneously represents a vulnerability that bacteria exploit through precise regulation of its three pathways: IRE1α‐XBP1, PERK‐eIF2α‐ATF4, and ATF6. Pathogens strategically manipulate UPR signaling through secreted virulence factors that target ER chaperones, disrupt calcium homeostasis, interfere with ER‐associated degradation, and modulate ER‐phagy. Rather than uniformly activating or inhibiting the UPR, bacteria selectively modulate specific pathways to establish favorable intracellular niches while disrupting normal physiological functions and dampening pro‐inflammatory and apoptotic signals.

Pharmacological targeting of UPR components represents a novel frontier in antimicrobial therapy. UPR modulatory compounds—including PERK inhibitors, IRE1α inhibitors, and ATF6 inhibitors—can disrupt pathogen hijacking of signaling pathways, restoring host cellular protein homeostasis and immune function. This represents a paradigm shift from conventional antimicrobials toward host‐directed therapies that exploit bacterial dependence on hijacked cellular machinery. However, challenges regarding target selectivity and safety profiles remain significant considerations for clinical development.

Future research should explore UPR‐modulating drug therapeutic potential in resolving persistent intracellular infections, particularly in conjunction with existing antimicrobial agents. Understanding bacterial UPR exploitation mechanisms will illuminate the molecular basis of infection and reveal promising targets for developing novel antibacterial therapies. The UPR thus represents a dynamic interface in host‐pathogen conflicts where bacterial adaptations reveal therapeutic vulnerabilities, opening avenues for innovative host‐directed interventions to combat antimicrobial resistance.

## Author Contributions

Enhui Dai and Dongjie Sun summarized the molecular mechanism of UPR and drafted the manuscript. Dongjie Sun and Yanxiao Zhao designed the images. Mengtao Zhang and Yifan Wu collected and organized the literature. Dongjie Sun and Jiabo Ding supervised the project and edited the manuscript. All authors contributed to this article and approved the submitted version.

## Funding

This work was funded by the Science and Technology Innovation Project of the Chinese Academy of Agricultural Sciences (CAAS‐CSLPDCP‐202403), Postdoctoral Fellowship Program of CPSF under Grant Number 2025 T180827 and GZB20250698.

## Conflicts of Interest

The authors declare no conflicts of interest.

## Data Availability

No datasets were generated or analyzed in the course of this study.

## References

[1] M. A. Brostrom and C. O. Brostrom , “Calcium Dynamics and Endoplasmic Reticular Function in the Regulation of Protein Synthesis: Implications for Cell Growth and Adaptability,” Cell Calcium 34 (2003): 345–363. 12909081 10.1016/s0143-4160(03)00127-1

[2] B. Kleizen and I. Braakman , “Protein Folding and Quality Control in the Endoplasmic Reticulum,” Current Opinion in Cell Biology 16 (2004): 343–349. 15261665 10.1016/j.ceb.2004.06.012

[3] I. Braakman and N. J. Bulleid , “Protein Folding and Modification in the Mammalian Endoplasmic Reticulum,” Annual Review of Biochemistry 80 (2011): 71–99. 10.1146/annurev-biochem-062209-093836 21495850

[4] J. Celli and R. M. Tsolis , “Bacteria, the Endoplasmic Reticulum and the Unfolded Protein Response: Friends or Foes?,” Nature Reviews Microbiology 13 (2015): 71–82. 25534809 10.1038/nrmicro3393 PMC4447104

[5] C. Hetz , F. Martinon , D. Rodriguez , and L. H. Glimcher , “The Unfolded Protein Response: Integrating Stress Signals Through the Stress Sensor IRE1α,” Physiological Reviews 91 (2011): 1219–1243. 22013210 10.1152/physrev.00001.2011

[6] B. He , “Viruses, Endoplasmic Reticulum Stress, and Interferon Responses,” Cell Death & Differentiation 13 (2006): 393–403. 16397582 10.1038/sj.cdd.4401833

[7] R. Liang , Y. Fu , G. Li , et al., “EP152R‐Mediated Endoplasmic Reticulum Stress Contributes to African Swine Fever Virus Infection via the PERK‐eIF2α Pathway,” FASEB Journal 38 (2024): e70187. 39560029 10.1096/fj.202400931RR

[8] Y. Wang , J. Li , H. Cao , et al., “African Swine Fever Virus Modulates the Endoplasmic Reticulum Stress‐ATF6‐Calcium Axis to Facilitate Viral Replication,” Emerging Microbes & Infections 13 (2024): 2399945. 39230190 10.1080/22221751.2024.2399945 PMC11441038

[9] C. Hetz , “The Unfolded Protein Response: Controlling Cell Fate Decisions Under ER Stress and Beyond,” Nature Reviews. Molecular Cell Biology 13 (2012): 89–102. 22251901 10.1038/nrm3270

[10] T. Rzymski , M. Milani , D. C. Singleton , and A. L. Harris , “Role of ATF4 in Regulation of Autophagy and Resistance to Drugs and Hypoxia,” Cell Cycle 8 (2009): 3838–3847. 19887912 10.4161/cc.8.23.10086

[11] S. Song , J. Tan , Y. Miao , and Q. Zhang , “Crosstalk of ER Stress‐Mediated Autophagy and ER‐Phagy: Involvement of UPR and the Core Autophagy Machinery,” Journal of Cellular Physiology 233 (2018): 3867–3874. 28777470 10.1002/jcp.26137

[12] H. Urra , E. Dufey , F. Lisbona , D. Rojas‐Rivera , and C. Hetz , “When ER Stress Reaches a Dead End,” Biochimica et Biophysica Acta (BBA) 1833 (2013): 3507–3517. 23988738 10.1016/j.bbamcr.2013.07.024

[13] J. Celli and J. P. Gorvel , “Organelle Robbery: *Brucella* Interactions With the Endoplasmic Reticulum,” Current Opinion in Microbiology 7 (2004): 93–97. 15036147 10.1016/j.mib.2003.11.001

[14] D. Pincus , M. W. Chevalier , T. Aragón , et al., “BiP Binding to the ER‐Stress Sensor Ire1 Tunes the Homeostatic Behavior of the Unfolded Protein Response,” PLoS Biology 8 (2010): e1000415. 20625545 10.1371/journal.pbio.1000415 PMC2897766

[15] A. Pandey , F. Lin , A. L. Cabello , et al., “Activation of Host IRE1α‐Dependent Signaling Axis Contributes the Intracellular Parasitism of *Brucella melitensis* ,” Frontiers in Cellular and Infection Microbiology 8 (2018): 103. 29732320 10.3389/fcimb.2018.00103 PMC5919948

[16] Y. Adachi , K. Yamamoto , T. Okada , H. Yoshida , A. Harada , and K. Mori , “ATF6 Is a Transcription Factor Specializing in the Regulation of Quality Control Proteins in the Endoplasmic Reticulum,” Cell Structure and Function 33 (2008): 75–89. 18360008 10.1247/csf.07044

[17] N. R. Guydosh , P. Kimmig , P. Walter , and R. Green , “Regulated Ire1‐Dependent mRNA Decay Requires No‐Go mRNA Degradation to Maintain Endoplasmic Reticulum Homeostasis in *S. pombe* ,” eLife 6 (2017): e29216. 28945192 10.7554/eLife.29216 PMC5650469

[18] D. Han , A. G. Lerner , L. Vande Walle , et al., “IRE1alpha Kinase Activation Modes Control Alternate Endoribonuclease Outputs to Determine Divergent Cell Fates,” Cell 138 (2009): 562–575. 19665977 10.1016/j.cell.2009.07.017 PMC2762408

[19] J. Wang , J. Y. Liu , K. Y. Shao , et al., “Porcine Reproductive and Respiratory Syndrome Virus Activates Lipophagy to Facilitate Viral Replication Through Downregulation of NDRG1 Expression,” Journal of Virology 93 (2019): e00526‐19. 31189711 10.1128/JVI.00526-19 PMC6694807

[20] S. E. Bettigole and L. Glimcher , “Endoplasmic Reticulum Stress in Immunity,” Annual Review of Immunology 33 (2015): 107–138. 10.1146/annurev-immunol-032414-112116 25493331

[21] W. Huang , Y. Gong , and L. Yan , “ER Stress, the Unfolded Protein Response and Osteoclastogenesis: A Review,” Biomolecules 13 (2023): 1050. 37509086 10.3390/biom13071050 PMC10377020

[22] H. Dong , N. M. Adams , Y. Xu , et al., “The IRE1 Endoplasmic Reticulum Stress Sensor Activates Natural Killer Cell Immunity in Part by Regulating c‐Myc,” Journal Nature Immunology 20 (2019): 865–878. 10.1038/s41590-019-0388-z PMC6588410 31086333

[23] S. Chen , J. Chen , X. Hua , et al., “The Emerging Role of XBP1 in Cancer,” Biomedicine & Pharmacotherapy 127 (2020): 110069. 32294597 10.1016/j.biopha.2020.110069

[24] H. P. Harding , Y. Zhang , H. Zeng , et al., “An Integrated Stress Response Regulates Amino Acid Metabolism and Resistance to Oxidative Stress,” Molecular Cell 11 (2003): 619–633. 12667446 10.1016/s1097-2765(03)00105-9

[25] H. P. Harding , Y. Zhang , A. Bertolotti , H. Zeng , and D. Ron , “Perk Is Essential for Translational Regulation and Cell Survival During the Unfolded Protein Response,” Molecular Cell 5 (2000): 897–904. 10882126 10.1016/s1097-2765(00)80330-5

[26] J. A. Choi and C. H. Song , “Insights Into the Role of Endoplasmic Reticulum Stress in Infectious Diseases,” Frontiers in Immunology 10 (2019): 3147. 32082307 10.3389/fimmu.2019.03147 PMC7005066

[27] R. Asada , S. Kanemoto , S. Kondo , A. Saito , and K. Imaizumi , “The Signalling From Endoplasmic Reticulum‐Resident bZIP Transcription Factors Involved in Diverse Cellular Physiology,” Journal of Biochemistry 149 (2011): 507–518. 21454302 10.1093/jb/mvr041

[28] K. Haze , H. Yoshida , H. Yanagi , T. Yura , and K. Mori , “Mammalian Transcription Factor ATF6 Is Synthesized as a Transmembrane Protein and Activated by Proteolysis in Response to Endoplasmic Reticulum Stress,” Molecular Biology of the Cell 10 (1999): 3787–3799. 10564271 10.1091/mbc.10.11.3787 PMC25679

[29] D. Senft and Z. A. Ronai , “UPR, Autophagy, and Mitochondria Crosstalk Underlies the ER Stress Response,” Trends in Biochemical Sciences 40 (2015): 141–148. 25656104 10.1016/j.tibs.2015.01.002 PMC4340752

[30] A. R. van Vliet , T. Verfaillie , and P. Agostinis , “New Functions of Mitochondria Associated Membranes in Cellular Signaling,” Biochimica et Biophysica Acta (BBA) 1843 (2014): 2253–2262. 24642268 10.1016/j.bbamcr.2014.03.009

[31] K. R. Bhattarai , T. A. Riaz , H. R. Kim , and H. J. Chae , “The Aftermath of the Interplay Between the Endoplasmic Reticulum Stress Response and Redox Signaling,” Experimental & Molecular Medicine 53 (2021): 151–167. 33558590 10.1038/s12276-021-00560-8 PMC8080639

[32] K. Arasaki , Y. Mikami , S. R. Shames , H. Inoue , Y. Wakana , and M. Tagaya , “Legionella Effector Lpg1137 Shuts Down ER‐Mitochondria Communication Through Cleavage of Syntaxin 17,” Nature Communications 8 (2017): 15406. 10.1038/ncomms15406 PMC5440676 28504273

[33] V. Prasad , “Transmission of Unfolded Protein Response—A Regulator of Disease Progression, Severity, and Spread in Virus Infections,” mBio 16 (2025): e0352224. 39772778 10.1128/mbio.03522-24 PMC11796368

[34] F. G. da Fonseca , Â. V. Serufo , T. L. Leão , and K. L. Lourenço , “Viral Infections and Their Ability to Modulate Endoplasmic Reticulum Stress Response Pathways,” Viruses 16 (2024): 1555. 39459886 10.3390/v16101555 PMC11512299

[35] M. H. Alshareef , E. L. Hartland , and K. McCaffrey , “Effectors Targeting the Unfolded Protein Response During Intracellular Bacterial Infection,” Microorganisms 9 (2021): 705. 33805575 10.3390/microorganisms9040705 PMC8065698

[36] C. G. Robinson and C. R. Roy , “Attachment and Fusion of Endoplasmic Reticulum With Vacuoles Containing *Legionella pneumophila* ,” Cellular Microbiology 8 (2006): 793–805. 16611228 10.1111/j.1462-5822.2005.00666.x

[37] T. Banerjee , A. Grabon , M. Taylor , and K. Teter , “cAMP‐Independent Activation of the Unfolded Protein Response by Cholera Toxin,” Infection and Immunity Journal 89 (2021): e00447‐20. 10.1128/IAI.00447-20 PMC7822150 33199355

[38] M. F. de Jong , T. Starr , M. G. Winter , et al., “Sensing of Bacterial Type IV Secretion via the Unfolded Protein Response,” mBio 4 (2013): e00418‐12. 23422410 10.1128/mBio.00418-12 PMC3624511

[39] C. N. Miller , E. P. Smith , J. A. Cundiff , et al., “A *Brucella* Type IV Effector Targets the COG Tethering Complex to Remodel Host Secretory Traffic and Promote Intracellular Replication,” Cell Host & Microbe 22 (2017): 317–329. 28844886 10.1016/j.chom.2017.07.017 PMC5599354

[40] J. A. Smith , M. Khan , D. D. Magnani , et al., “ *Brucella* Induces an Unfolded Protein Response via TcpB That Supports Intracellular Replication in Macrophages,” PLoS Pathogens 9 (2013): e1003785. 24339776 10.1371/journal.ppat.1003785 PMC3855547

[41] J. B. Luizet , J. Raymond , T. L. S. Lacerda , et al., “The *Brucella* Effector BspL Targets the ER‐Associated Degradation (ERAD) Pathway and Delays Bacterial Egress From Infected Cells,” Proceedings of the National Academy of Sciences of the United States of America 118 (2021): e2105324118. 34353909 10.1073/pnas.2105324118 PMC8364137

[42] S. Grover , T. Sharma , Y. Singh , et al., “The PGRS Domain of *Mycobacterium tuberculosis* PE_PGRS Protein Rv0297 Is Involved in Endoplasmic Reticulum Stress‐Mediated Apoptosis Through Toll‐Like Receptor 4,” mBio 9 (2018): e01017‐18. 29921671 10.1128/mBio.01017-18 PMC6016250

[43] J. A. Choi , Y. J. Lim , S. N. Cho , et al., “Mycobacterial HBHA Induces Endoplasmic Reticulum Stress‐Mediated Apoptosis Through the Generation of Reactive Oxygen Species and Cytosolic Ca2+ in Murine Macrophage RAW 264.7 Cells,” Cell Death and Disease 4 (2013): e957. 24336077 10.1038/cddis.2013.489 PMC3877560

[44] P. Xu , J. Tang , and Z. G. He , “Induction of Endoplasmic Reticulum Stress by CdhM Mediates Apoptosis of Macrophage During *Mycobacterium tuberculosis* Infection,” Frontiers in Cellular and Infection Microbiology 12 (2022): 877265. 35444960 10.3389/fcimb.2022.877265 PMC9013901

[45] N. U. Ibe , A. Subramanian , and S. Mukherjee , “Non‐Canonical Activation of the ER Stress Sensor ATF6 by *Legionella pneumophila* Effectors,” Life Science Alliance 4 (2021): e202101247. 34635501 10.26508/lsa.202101247 PMC8507491

[46] H. Pillich , M. Loose , K. P. Zimmer , and T. Chakraborty , “Activation of the Unfolded Protein Response by *Listeria monocytogenes* ,” Cellular Microbiology 14 (2012): 949–964. 22321539 10.1111/j.1462-5822.2012.01769.x

[47] M. S. Simpson , H. De Luca , S. Cauthorn , et al., “IRE1α Recognizes a Structural Motif in Cholera Toxin to Activate an Unfolded Protein Response,” Journal of Cell Biology 223 (2024): e202402062. 38578285 10.1083/jcb.202402062 PMC10996581

[48] J. Celli , C. de Chastellier , D. M. Franchini , J. Pizarro‐Cerda , E. Moreno , and J. P. Gorvel , “ *Brucella* Evades Macrophage Killing via VirB‐Dependent Sustained Interactions With the Endoplasmic Reticulum,” Journal of Experimental Medicine 198 (2003): 545–556. 12925673 10.1084/jem.20030088 PMC2194179

[49] C. Hetz , K. Zhang , and R. J. Kaufman , “Mechanisms, Regulation and Functions of the Unfolded Protein Response,” Nature Reviews Molecular Cell Biology 21 (2020): 421–438. 32457508 10.1038/s41580-020-0250-z PMC8867924

[50] E. P. Smith , A. Cotto‐Rosario , E. Borghesan , K. Held , C. N. Miller , and J. Celli , “Epistatic Interplay Between Type IV Secretion Effectors Engages the Small GTPase Rab2 in the *Brucella* Intracellular Cycle,” mBio 11 (2020): e03350‐19. 32234817 10.1128/mBio.03350-19 PMC7157780

[51] K. Byrnes , S. Blessinger , N. T. Bailey , R. Scaife , G. Liu , and B. Khambu , “Therapeutic Regulation of Autophagy in Hepatic Metabolism,” Acta Pharmaceutica Sinica B 12 (2022): 33–49. 35127371 10.1016/j.apsb.2021.07.021 PMC8799888

[52] Z. Sun and J. L. Brodsky , “Protein Quality Control in the Secretory Pathway,” Journal of Cell Biology 218 (2019): 3171–3187. 31537714 10.1083/jcb.201906047 PMC6781448

[53] H. H. Choi , D. M. Shin , G. Kang , et al., “Endoplasmic Reticulum Stress Response Is Involved in *Mycobacterium tuberculosis* Protein ESAT‐6‐Mediated Apoptosis,” FEBS Letters 584 (2010): 2445–2454. 20416295 10.1016/j.febslet.2010.04.050

[54] S. Kambarev , E. Borghesan , C. N. Miller , S. Myeni , and J. Celli , “The *Brucella abortus* Type IV Effector BspA Inhibits MARCH6‐Dependent ERAD to Promote Intracellular Growth,” Infection and Immunity 91 (2023): e0013023. 37129527 10.1128/iai.00130-23 PMC10187129

[55] A. Hempstead and R. R. DIsberg , “Inhibition of Host Cell Translation Elongation by *Legionella pneumophila* Blocks the Host Cell Unfolded Protein Response,” Proceedings of the National Academy of Sciences of the United States of America 112 (2015): E6790–E6797. 26598709 10.1073/pnas.1508716112 PMC4679008

[56] S. Treacy‐Abarca and S. Mukherjee , “ *Legionella* Suppresses the Host Unfolded Protein Response via Multiple Mechanisms,” Nature Communications 6 (2015): 7887. 10.1038/ncomms8887 PMC4519984 26219498

[57] K. Mochida and H. Nakatogawa , “ER‐Phagy: Selective Autophagy of the Endoplasmic Reticulum,” EMBO Reports 23 (2022): e55192. 35758175 10.15252/embr.202255192 PMC9346472

[58] J. C. Y. Christianson and Y. Ye , “Cleaning Up in the Endoplasmic Reticulum: Ubiquitin in Charge,” Nature Structural & Molecular Biology 21 (2014): 325–335. 10.1038/nsmb.2793 PMC9397582 24699081

[59] W. Chen , H. Mao , L. Chen , and L. Li , “The Pivotal Role of FAM134B in Selective ER‐Phagy and Diseases,” Biochimica et Biophysica Acta (BBA) 1869 (2022): 119277. 10.1016/j.bbamcr.2022.119277 35477002

[60] D. Gatica , R. M. Alsaadi , R. El Hamra , et al., “The ER‐Phagy Receptor FAM134B Is Targeted by *Salmonella typhimurium* to Promote Infection,” Nature Communications 16 (2025): 2923. 10.1038/s41467-025-58035-7 PMC11937434 40133256

[61] H. Chino and N. Mizushima , “ER‐Phagy: Quality and Quantity Control of the Endoplasmic Reticulum by Autophagy,” Cold Spring Harbor Perspectives in Biology 15 (2023): a041256. 35940904 10.1101/cshperspect.a041256 PMC9808580

[62] S. Li , R. Yan , J. Xu , et al., “A New Type of ERGIC‐ERES Membrane Contact Mediated by TMED9 and SEC12 Is Required for Autophagosome Biogenesis,” Cell Research 32 (2022): 119–138. 34561617 10.1038/s41422-021-00563-0 PMC8461442

[63] N. Sharma , M. Shariq , N. Quadir , et al., “ *Mycobacterium tuberculosis* Protein PE6 (Rv0335c), a Novel TLR4 Agonist, Evokes an Inflammatory Response and Modulates the Cell Death Pathways in Macrophages to Enhance Intracellular Survival,” Frontiers in Immunology 12 (2021): 696491. 34322125 10.3389/fimmu.2021.696491 PMC8311496

[64] N. K. Saini , A. Baena , T. W. Ng , et al., “Suppression of Autophagy and Antigen Presentation by *Mycobacterium tuberculosis* PE_PGRS47,” Nature Microbiology 1 (2016): 16133. 10.1038/nmicrobiol.2016.133 PMC5662936 27562263

[65] E. J. Strong , T. W. Ng , S. A. Porcelli , and S. Lee , “ *Mycobacterium tuberculosis* PE_PGRS20 and PE_PGRS47 Proteins Inhibit Autophagy by Interaction With Rab1A,” mSphere 6 (2021): e0054921. 34346699 10.1128/mSphere.00549-21 PMC8386380

[66] M. Shariq , N. Quadir , A. Alam , et al., “The Exploitation of Host Autophagy and Ubiquitin Machinery by *Mycobacterium tuberculosis* in Shaping Immune Responses and Host Defense During Infection,” Autophagy 19 (2023): 3–23. 35000542 10.1080/15548627.2021.2021495 PMC9809970

[67] R. Mukherjee , A. Bhattacharya , I. Tomaskovic , et al., “Phosphoribosyl Ubiquitination of SNARE Proteins Regulates Autophagy During Legionella Infection,” EMBO Journal 44 (2025): 4252–4279. 40506485 10.1038/s44318-025-00483-4 PMC12317070

[68] A. Yang , S. Pantoom , and Y. W. Wu , “Elucidation of the Anti‐Autophagy Mechanism of the Legionella Effector RavZ Using Semisynthetic LC3 Proteins,” eLife 6 (2017): e23905. 28395732 10.7554/eLife.23905 PMC5388539

[69] S. M. Oh , Y. J. Lim , J. A. Choi , et al., “TNF‐α‐Mediated ER Stress Causes Elimination of *Mycobacterium fortuitum* Reservoirs by Macrophage Apoptosis,” FASEB Journal 32 (2018): 3993–4003. 29481309 10.1096/fj.201701407R

[70] Z. Li , S. Wang , J. Han , et al., “Deletion of *Brucella* Transcriptional Regulator GntR10 Regulated the Expression of Quorum Sensing System and Type IV Secretion System Effectors, Which Affected the Activation of NF‐κB,” Journal of Proteomics 283‐284 (2023): 104938. 10.1016/j.jprot.2023.104938 37230328

[71] M. H. Smith , H. L. Ploegh , and J. S. Weissman , “Road to Ruin: Targeting Proteins for Degradation in the Endoplasmic Reticulum,” Science 334 (2011): 1086–1090. 22116878 10.1126/science.1209235 PMC3864754

[72] C. Atkins , Q. Liu , E. Minthorn , et al., “Characterization of a Novel PERK Kinase Inhibitor With Antitumor and Antiangiogenic Activity,” Cancer Research 73 (2013): 1993–2002. 23333938 10.1158/0008-5472.CAN-12-3109

[73] J. Krishnamoorthy , K. Rajesh , F. Mirzajani , P. Kesoglidou , A. I. Papadakis , and A. E. Koromilas , “Evidence for eIF2α Phosphorylation‐Independent Effects of GSK2656157, a Novel Catalytic Inhibitor of PERK With Clinical Implications,” Cell Cycle 13 (2014): 801–806. 24401334 10.4161/cc.27726 PMC3979916

[74] D. Rojas‐Rivera , T. Delvaeye , R. Roelandt , et al., “When PERK Inhibitors Turn Out to be New Potent RIPK1 Inhibitors: Critical Issues on the Specificity and Use of GSK2606414 and GSK2656157,” Cell Death and Differentiation 24 (2017): 1100–1110. 28452996 10.1038/cdd.2017.58 PMC5442476

[75] B. Ma , X. Nie , L. Liu , et al., “GSK2656157, a PERK Inhibitor, Alleviates Pyroptosis of Macrophages Induced by *Mycobacterium bacillus* Calmette‐Guerin Infection,” International Journal of Molecular Sciences 24 (2023): 16239. 38003429 10.3390/ijms242216239 PMC10671627

[76] S. H. E. Kaufmann , A. Dorhoi , R. S. Hotchkiss , and R. Bartenschlager , “Host‐Directed Therapies for Bacterial and Viral Infections,” Nature Reviews Drug Discovery 17 (2018): 35–56. 28935918 10.1038/nrd.2017.162 PMC7097079

[77] A. F. Zyryanova , K. Kashiwagi , C. Rato , et al., “ISRIB Blunts the Integrated Stress Response by Allosterically Antagonising the Inhibitory Effect of Phosphorylated eIF2 on eIF2B,” Molecular Cell 81 (2021): 88–103. 33220178 10.1016/j.molcel.2020.10.031 PMC7837216

[78] R. Ghosh , L. Wang , E. S. Wang , et al., “Allosteric Inhibition of the IRE1α RNase Preserves Cell Viability and Function During Endoplasmic Reticulum Stress,” Cell 158 (2014): 534–548. 25018104 10.1016/j.cell.2014.07.002 PMC4244221

[79] I. Papandreou , N. C. Denko , M. Olson , et al., “Identification of an Ire1alpha Endonuclease Specific Inhibitor With Cytotoxic Activity Against Human Multiple Myeloma,” Blood 117 (2011): 1311–1314. 21081713 10.1182/blood-2010-08-303099 PMC3056474

[80] D. J. Maly and F. R. Papa , “Druggable Sensors of the Unfolded Protein Response,” Nature Chemical Biology 10 (2014): 892–901. 25325700 10.1038/nchembio.1664 PMC4664160

[81] W. Zhang , M. Wang , K. Gao , et al., “Pharmacologic IRE1α Kinase Inhibition Alleviates Aortic Dissection by Decreasing Vascular Smooth Muscle Cells Apoptosis,” International Journal of Biological Sciences 18 (2022): 1053–1064. 35173538 10.7150/ijbs.63593 PMC8771832

[82] Z. Y. Zhou , L. Wu , Y. F. Liu , et al., “IRE1α: From the Function to the Potential Therapeutic Target in Atherosclerosis,” Molecular and Cellular Biochemistry 479 (2024): 1079–1092. 37310588 10.1007/s11010-023-04780-6

[83] E. A. Blackwood , K. Azizi , D. J. Thuerauf , et al., “Pharmacologic ATF6 Activation Confers Global Protection in Widespread Disease Models by Reprograming Cellular Proteostasis,” Nature Communications 10 (2019): 187. 10.1038/s41467-018-08129-2 PMC6331617 30643122

[84] C. M. Gallagher , C. Garri , E. L. Cain , et al., “Ceapins Are a New Class of Unfolded Protein Response Inhibitors, Selectively Targeting the ATF6α Branch,” eLife 5 (2016): e11878. 27435960 10.7554/eLife.11878 PMC4954757

[85] J. M. D. Grandjean and R. L. Wiseman , “Small Molecule Strategies to Harness the Unfolded Protein Response: Where Do We Go From Here?,” Journal of Biological Chemistry 295 (2020): 15692–15711. 32887796 10.1074/jbc.REV120.010218 PMC7667976

[86] J. T. Isaacs , W. N. Brennen , S. B. Christensen , and S. R. Denmeade , “Mipsagargin: The Beginning‐Not the End‐Of Thapsigargin Prodrug‐Based Cancer Therapeutics,” Molecules 26 (2021): 7469. 34946547 10.3390/molecules26247469 PMC8707208

[87] N. I. Agalakova , “Modulation of Endoplasmic Reticulum Stress in Experimental Anti‐Cancer Therapy,” International Journal of Molecular Sciences 26 (2025): 6407. 40650182 10.3390/ijms26136407 PMC12250421

[88] Z. G. Zheng , S. T. Zhu , H. M. Cheng , et al., “Discovery of a Potent SCAP Degrader That Ameliorates HFD‐Induced Obesity, Hyperlipidemia and Insulin Resistance via an Autophagy‐Independent Lysosomal Pathway,” Journal of Autophagy 17 (2021): 1592–1613. 32432943 10.1080/15548627.2020.1757955 PMC8354609

[89] A. Jaskulska , A. E. Janecka , and K. Gach‐Janczak , “Thapsigargin‐From Traditional Medicine to Anticancer Drug,” International Journal of Molecular Sciences 22 (2020): 4. 33374919 10.3390/ijms22010004 PMC7792614

[90] C. Hetz , E. Chevet , and H. P. Harding , “Targeting the Unfolded Protein Response in Disease,” Nature Reviews Drug Discovery 12 (2013): 703–719. 23989796 10.1038/nrd3976

[91] J. A. Moreno , M. Halliday , C. Molloy , et al., “Oral Treatment Targeting the Unfolded Protein Response Prevents Neurodegeneration and Clinical Disease in Prion‐Infected Mice,” Science Translational Medicine 5 (2013): 206ra138. 10.1126/scitranslmed.3006767 24107777

[92] A. Zumla , M. Rao , R. S. Wallis , et al., “Host‐Directed Therapies for Infectious Diseases: Current Status, Recent Progress, and Future Prospects,” Lancet Infectious Diseases 16 (2016): e47–e63. 27036359 10.1016/S1473-3099(16)00078-5 PMC7164794

